# Thoracoscopic Esophagectomy for Metachronous Esophageal Cancer after Heller-Dor Surgery for Achalasia with Marked Esophageal Dilatation: A Case Report

**DOI:** 10.70352/scrj.cr.26-0566

**Published:** 2026-10-16

**Authors:** Byonggu An, Tetsuya Abe, Yasumitsu Oe, Hiromitsu Ban, Toru Imagami, Akira Sogawa, Nobuyuki Takao, Takashi Matsunaga, Akiyoshi Mizumoto, Shizuki Takemura, Atsushi Hirata, Takuma Kawaguchi, Toru Miyake, Takeshi Togawa

**Affiliations:** 1 Department of Digestive Surgery, Omi Medical Center, Kusatsu, Shiga, Japan; 2 Department of Surgery, Shiga University of Medical Science, Kusatsu, Shiga, Japan; 3 Department of Gastroenterological Surgery, Aichi Cancer Center Hospital, Nagoya, Aichi, Japan; 4 Department of Gastroenterology, Omi Medical Center, Kusatsu, Shiga, Japan; 5 Department of Diagnostic Pathology, Omi Medical Center, Kusatsu, Shiga, Japan

**Keywords:** achalasia, Heller-Dor procedure, metachronous esophageal cancer, endoscopic submucosal dissection (ESD), thoracoscopic surgery

## Abstract

**INTRODUCTION:**

Achalasia is associated with an increased risk of esophageal squamous cell carcinoma even after definitive surgical treatment. Heller-Dor surgery is an established surgical treatment for achalasia; however, marked esophageal dilatation may persist despite successful symptom control and pose technical challenges during subsequent esophagectomy.

**CASE PRESENTATION:**

A 73-year-old man with a history of Heller-Dor surgery for achalasia and prior endoscopic submucosal dissection (ESD) for esophageal squamous cell carcinoma developed a metachronous lesion at a different esophageal site. Histopathological examination after ESD demonstrated deep submucosal and vascular invasion, warranting additional curative treatment. Preoperative imaging showed predominant upper thoracic esophageal dilatation with a maximum diameter of 56 mm, whereas gastric deformity was minimal. The patient underwent prone-position video-assisted thoracoscopic subtotal esophagectomy with 3-field lymphadenectomy and retrosternal gastric conduit reconstruction. Intraoperatively, venous engorgement required meticulous dissection, while marked upper thoracic esophageal dilatation restricted the operative workspace around the left recurrent laryngeal nerve. An internal organ retractor provided stable esophageal traction with flexible repositioning of the traction point, facilitating adequate exposure. Despite intra-abdominal adhesions from the previous surgery, gastric conduit reconstruction was successfully completed as anticipated from the preoperative assessment of gastric anatomy. Final histopathological examination revealed no residual carcinoma or lymph node metastasis. The postoperative course was uneventful, and the patient remained recurrence-free at 2 years.

**CONCLUSIONS:**

Long-term endoscopic surveillance remains important after surgical treatment for achalasia. In patients requiring subsequent esophagectomy, preoperative assessment of the degree and distribution of esophageal dilatation may help anticipate technical difficulty, while appropriate repositionable traction may facilitate operative exposure. Careful assessment of gastric anatomy may also help determine the feasibility of gastric conduit reconstruction after Heller-Dor surgery.

## Abbreviations

ESD
: endoscopic submucosal dissection
UICC
: Union for International Cancer Control

## INTRODUCTION

Achalasia is a rare primary esophageal motility disorder characterized by impaired relaxation of the lower esophageal sphincter and loss of esophageal peristalsis, leading to progressive dysphagia and esophageal dilatation.^1)^ Although the Heller myotomy with anterior fundoplication (Heller-Dor procedure) is widely recognized as an effective surgical treatment for the control of symptoms, several studies have reported that even after definitive therapy, patients with achalasia have a persistent long-term risk of developing esophageal squamous cell carcinoma.^2,3)^ Chronic stasis-induced inflammation, bacterial overgrowth, and mucosal degeneration are believed to contribute to carcinogenesis in the affected esophagus.^4,5)^

Esophagectomy for esophageal cancer arising after treatment for achalasia may present specific technical challenges. Persistent esophageal dilatation after Heller-Dor surgery may interfere with adequate operative exposure and complicate thoracoscopic dissection.^6)^ In addition, previous surgical treatment for achalasia, including myotomy and fundoplication, may result in adhesions and anatomical alterations that should be considered when planning subsequent esophagectomy and reconstruction.^6)^ However, the technical implications of persistent esophageal dilatation and previous Heller-Dor surgery for subsequent esophagectomy have rarely been described.^6,7)^

Herein, we report a rare case of metachronous esophageal squamous cell carcinoma developing 15 years after Heller-Dor surgery for achalasia and treated with thoracoscopic subtotal esophagectomy after ESD. We focus on the technical challenges associated with persistent esophageal dilatation, strategies for maintaining adequate operative exposure, and preoperative assessment of the feasibility of gastric conduit reconstruction after Heller-Dor surgery.

## CASE PRESENTATION

A 73-year-old man with a history of open Heller-Dor surgery for achalasia at age 58 and prior ESD for esophageal squamous cell carcinoma at age 67 was referred to our institution after routine surveillance esophagogastroduodenoscopy revealed a new lesion at a distinct location in the middle thoracic esophagus. Endoscopy identified a 20-mm type 0-IIc lesion approximately 30 cm from the incisors, and ESD was subsequently performed (**Fig. 1A**, **1B**). Histopathological examination revealed a moderately differentiated squamous cell carcinoma with submucosal invasion (SM2) measuring 425 μm beyond the muscularis mucosae (pT1b-SM2), type INFb, Ly0, V1, pHM0, and pVM0 (**Fig. 1C**, **1D**). Although the horizontal and vertical margins were negative, additional curative treatment was indicated because of deep submucosal and vascular invasion (V1). The patient was subsequently referred to our surgical department for further management. Contrast-enhanced CT showed no evidence of regional lymph node involvement or distant metastatic disease. CT also demonstrated marked esophageal dilatation, particularly in the upper mediastinum (**Fig. 2A**–**2D**), while coronal images showed the previous fundoplication with only minimal gastric deformity after the Heller-Dor procedure (**Fig. 3A**, **3B**). According to the UICC classification, 8th edition, the clinical stage was cT1bN0M0, corresponding to Stage I esophageal squamous cell carcinoma.

**Fig. 1:**
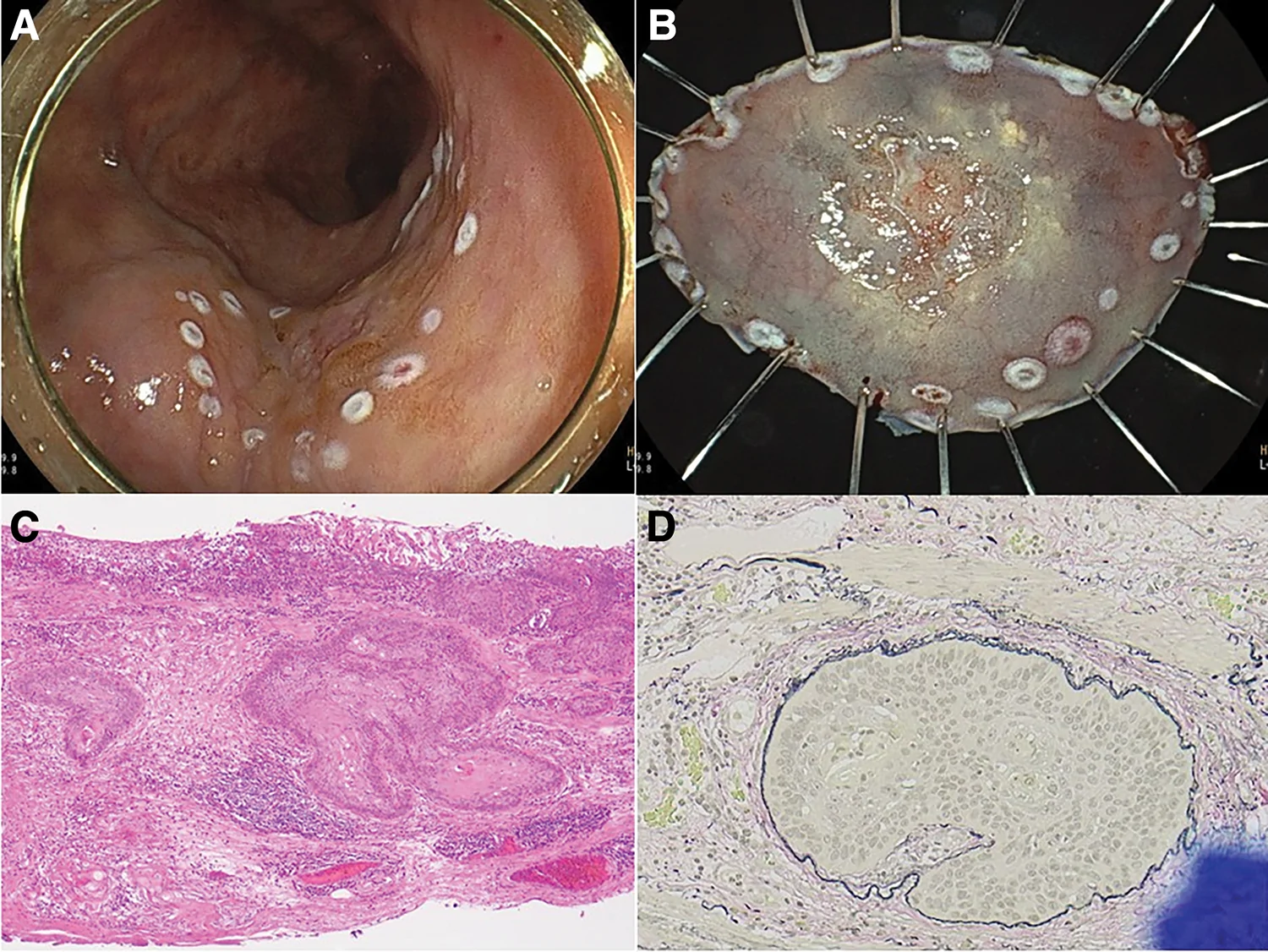
Endoscopic and histopathological findings of the metachronous esophageal squamous cell carcinoma. (**A**) Endoscopic view showing the lesion with marking dots before ESD. (**B**) En bloc ESD specimen pinned to a board for pathological evaluation. (**C**) Histopathological examination demonstrating moderately differentiated squamous cell carcinoma with SM2 (425 μm beyond the muscularis mucosae; pT1b-SM2). Hematoxylin and eosin stain, ×400. (**D**) Elastica van Gieson-stained section of the ESD specimen demonstrating V1, ×1000. ESD, endoscopic submucosal dissection

**Fig. 2:**
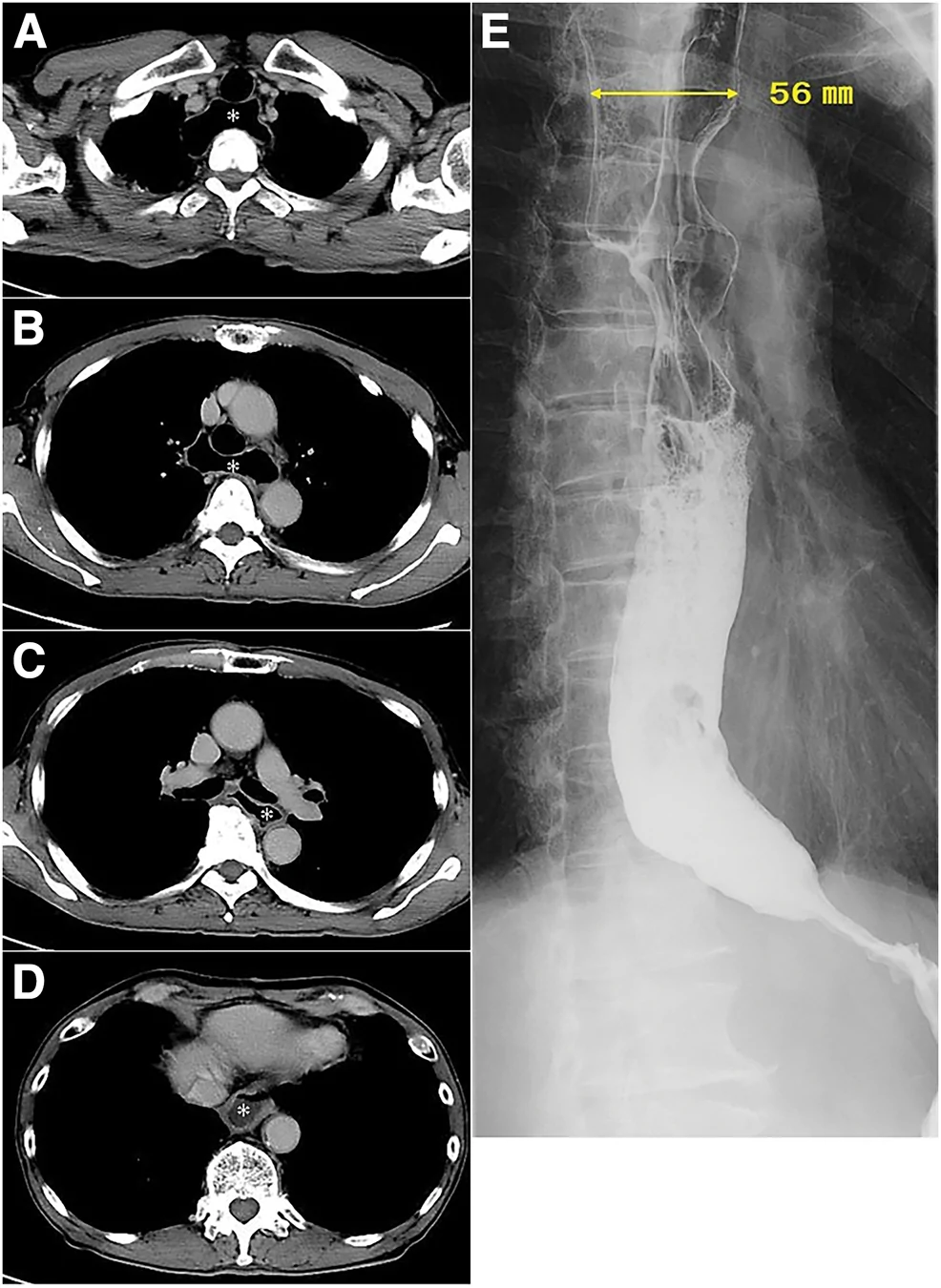
Preoperative imaging findings. (**A**–**D**) Axial contrast-enhanced CT images demonstrating marked esophageal dilatation (asterisks), which was particularly prominent in the upper mediastinum. No evidence of metastatic regional lymph nodes or distant metastatic disease was identified. (**E**) Upper gastrointestinal contrast study showing St achalasia with a maximum esophageal diameter of 56 mm in the upper thoracic esophagus, corresponding to Grade II esophageal dilatation. St, straight-type

**Fig. 3:**
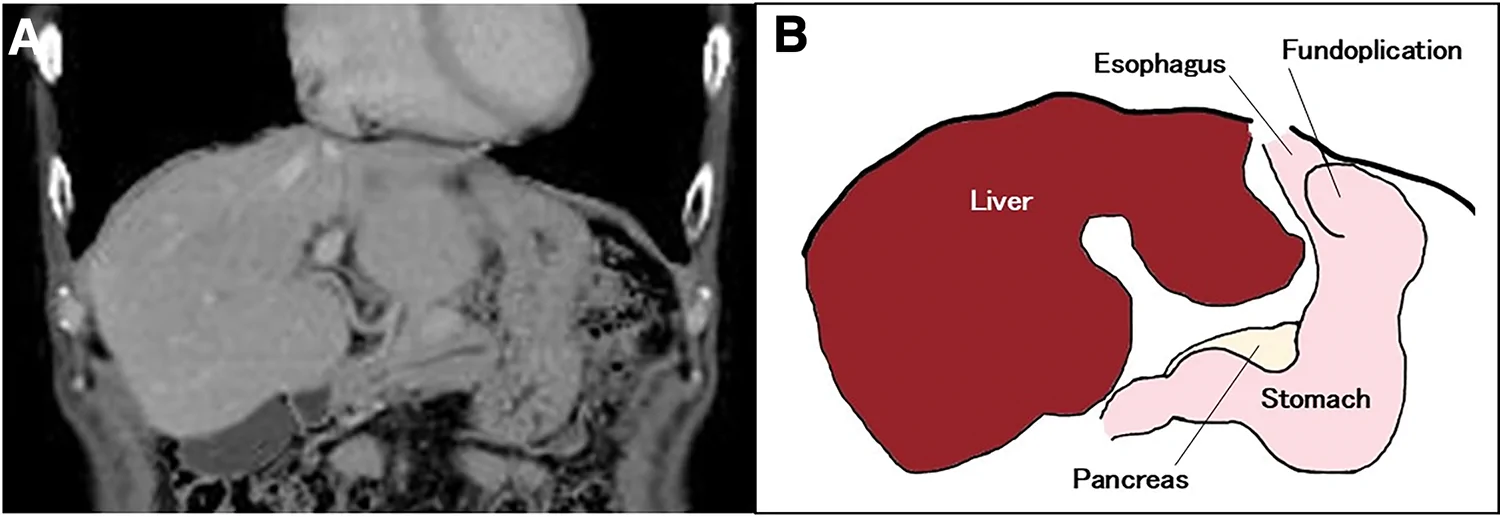
Preoperative assessment of gastric anatomy after Heller-Dor surgery. (**A**) Coronal CT image showing the previous fundoplication with only minimal gastric deformity after Heller-Dor surgery. (**B**) Schematic illustration of the corresponding anatomy, showing the anatomical relationship among the esophagus, fundoplication, stomach, liver, and pancreas.

An upper gastrointestinal contrast study demonstrated straight-type (St) achalasia with a maximum esophageal diameter of 56 mm in the upper thoracic esophagus. According to the Japanese classification of esophageal achalasia, the esophageal dilatation was classified as Grade II (**Fig. 2E**).^8)^

Both additional surgical resection and definitive chemoradiotherapy were considered because of the deep SM2 and V1 identified in the ESD specimen. The patient had a preserved performance status and preferred surgery because of concern about further metachronous lesions after having undergone ESD twice; therefore, additional esophagectomy with lymphadenectomy was selected as curative treatment. Given the limited gastric deformity observed on preoperative CT, the stomach was considered the primary option for reconstruction.

The patient underwent prone-position video-assisted thoracoscopic subtotal esophagectomy with 3-field lymphadenectomy, retrosternal gastric conduit reconstruction, and feeding jejunostomy placement. Surgical dissection was initiated in the middle and lower mediastinum. Prominent venous engorgement was observed along the middle and lower thoracic esophagus, requiring meticulous dissection to avoid vascular injury and bleeding (**Fig. 4A**). The markedly dilated esophagus restricted the operative workspace during upper mediastinal lymphadenectomy, particularly around the left recurrent laryngeal nerve (**Fig. 4B**). The Aesculap Internal Organ Retractor (Aesculap AG, Tuttlingen, Baden-Württemberg, Germany) was employed to provide stable esophageal traction and improve operative exposure (**Fig. 4C**). During the abdominal stage, adhesions from the previous surgery were encountered; however, gastric deformity was minimal, as anticipated from the preoperative CT findings, and gastric conduit construction was successfully completed. The total operative time was 583 min, including 301 min for the thoracic procedure, and the estimated blood loss was 360 mL.

**Fig. 4:**
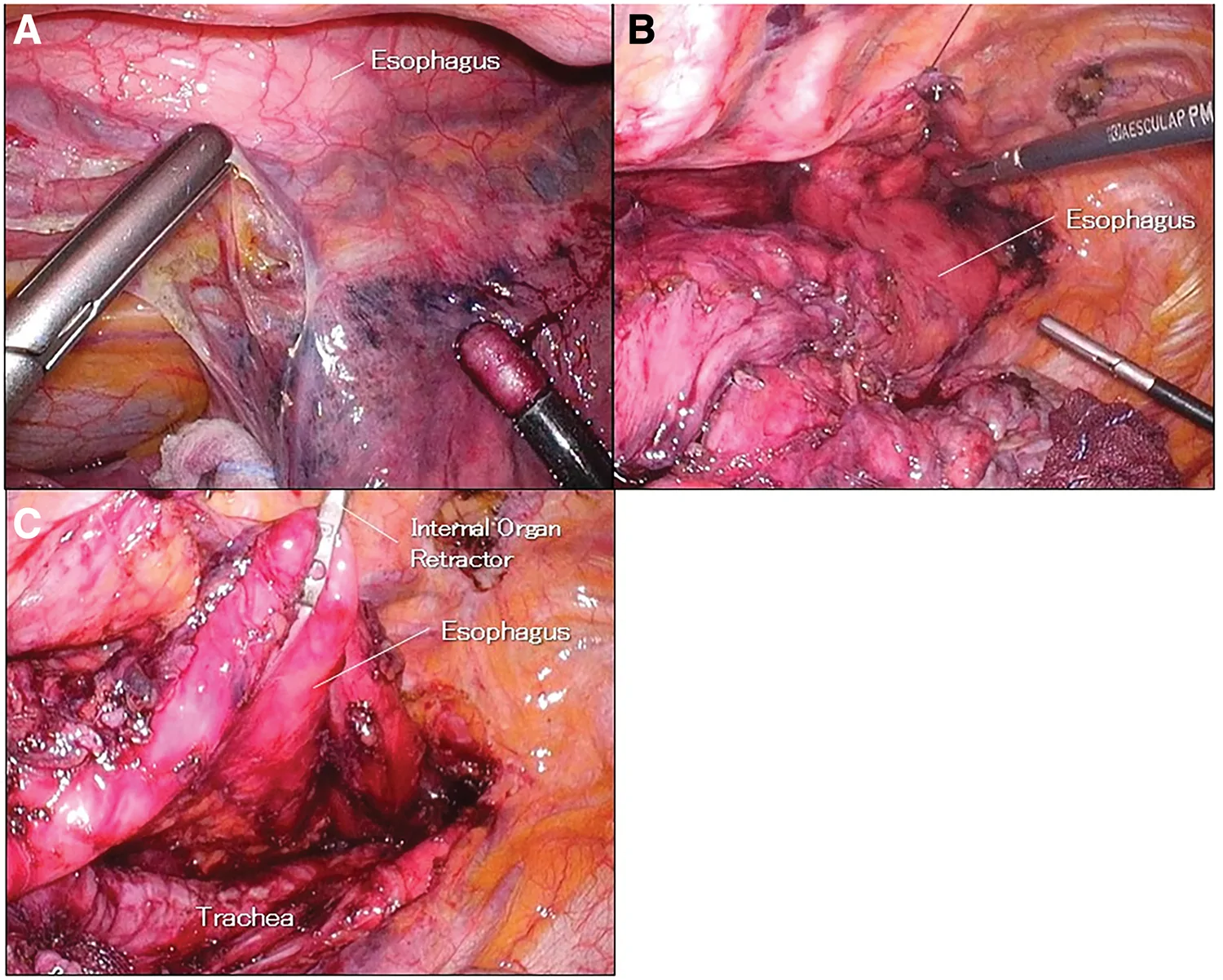
Intraoperative findings during thoracoscopic esophagectomy. (**A**) Prominent venous engorgement surrounding the middle and lower thoracic esophagus. (**B**) Markedly dilated esophagus in the upper mediastinum, resulting in a restricted operative field. (**C**) The dilated esophagus was retracted by an internal organ retractor, which provided stable traction and facilitated exposure during upper mediastinal dissection.

The resected specimen showed an ESD scar without residual carcinoma (**Fig. 5A**, **5B**), and no lymph node metastasis was observed. Based on the histopathological findings of the preceding ESD specimen and the esophagectomy specimen, the final pathological stage was pT1b(SM2)N0(0/80)M0, corresponding to Stage IB according to the UICC classification, 8th edition. Additional histopathological examination confirmed the absence of ganglion cells in the Auerbach plexus region, consistent with achalasia (**Fig. 5A**, **5C**). The previous Heller-Dor myotomy site was also identified, where the gastric wall used for fundoplication covered the esophageal muscle incision (**Fig. 5D**).

**Fig. 5:**
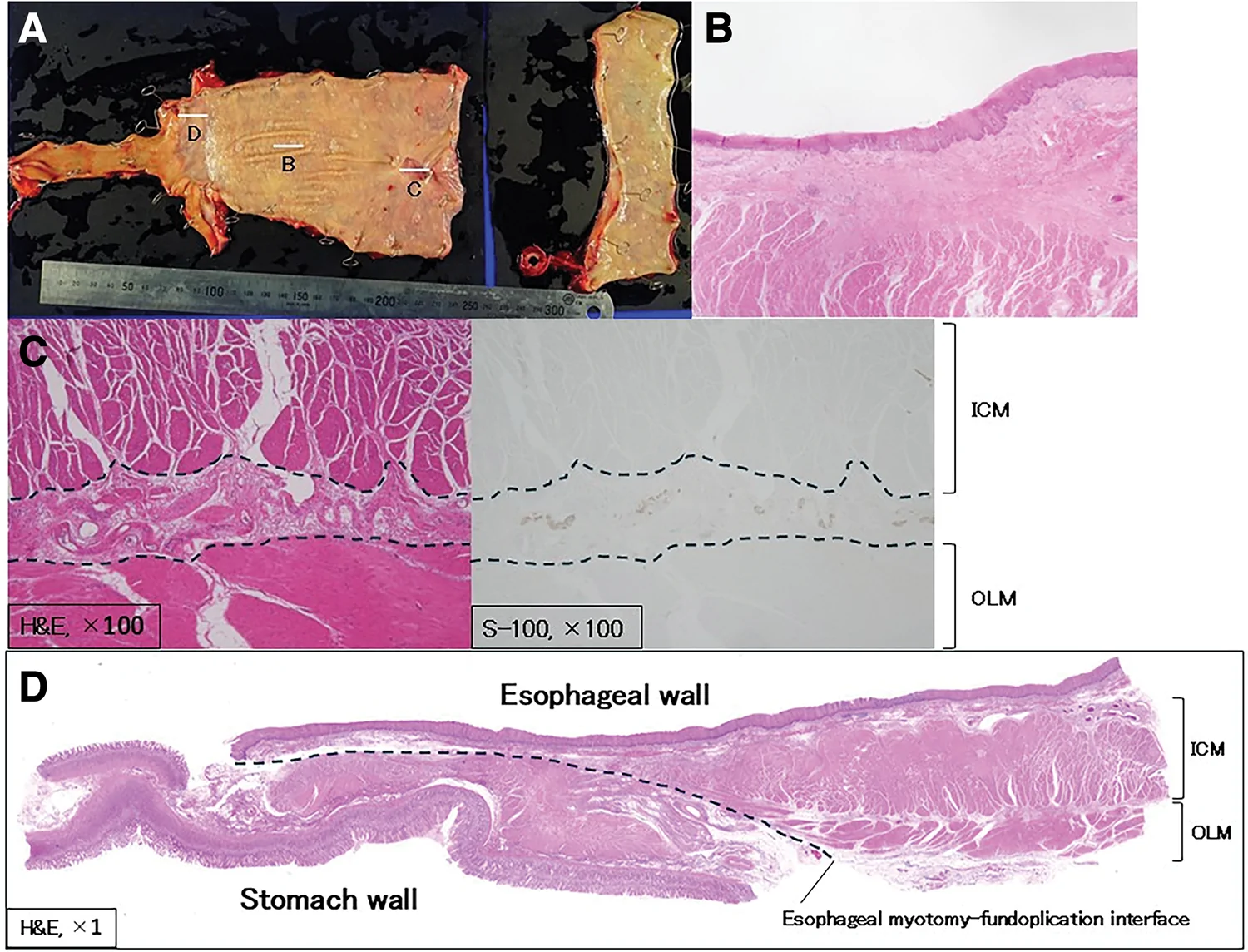
Histopathological findings of the esophagectomy specimen. (**A**) Macroscopic appearance of the resected specimen. White lines indicate the areas sampled for histopathological examination. (**B**) Histopathological examination of the ESD scar showing no residual carcinoma. H&E staining, original magnification ×20. (**C**) H&E staining (left) showing absence of ganglion cells in the Auerbach plexus region between the inner circular and outer longitudinal muscle layers, and S-100 immunohistochemical staining (right) of the corresponding region. Original magnification ×100. (**D**) Histopathological examination of the previous Heller-Dor myotomy site, showing the gastric wall covering the esophageal muscle incision at the myotomy–fundoplication interface. H&E staining, original magnification ×1. ESD, endoscopic submucosal dissection; H&E, hematoxylin and eosin; ICM, inner circular muscle; OLM, outer longitudinal muscle

The postoperative course was uneventful, with no recurrent laryngeal nerve palsy, anastomotic leakage, pneumonia, or other major postoperative complications. The patient was discharged on POD 23 and remained recurrence-free at the 2-year follow-up.

## DISCUSSION

This case provides 3 practical lessons for surgeons managing esophageal cancer in patients with previously treated achalasia. First, the risk of esophageal squamous cell carcinoma may persist long after Heller-Dor surgery despite successful symptom control, underscoring the need for long-term endoscopic surveillance.^6,9)^ Second, marked esophageal dilatation, particularly when predominant in the upper thoracic esophagus, may restrict the operative workspace around the left recurrent laryngeal nerve and increase the technical difficulty of upper mediastinal lymphadenectomy. In such cases, appropriate esophageal traction and flexible repositioning of the traction point may help maintain adequate operative exposure. Third, a gastric conduit may remain feasible after Heller-Dor surgery when substantial gastric deformity is absent.

Achalasia is associated with an increased risk of esophageal squamous cell carcinoma even after therapeutic intervention.^2)^ In the present patient, esophageal squamous cell carcinoma developed 9 years after Heller-Dor surgery, followed by a metachronous lesion at a different site 15 years after surgery despite sustained symptom control, supporting the importance of long-term endoscopic surveillance.^2,6,9)^

The principal technical challenge in the present case was thoracoscopic esophagectomy in the setting of persistent esophageal dilatation after Heller-Dor surgery. During mediastinal dissection, prominent vascular engorgement was observed around the middle and lower thoracic esophagus. Such vascular changes may have reflected longstanding esophageal remodeling associated with achalasia and chronic esophageal dilatation, potentially increasing the technical complexity of thoracoscopic dissection. Predominant dilatation of the upper thoracic esophagus further restricted the operative workspace in the upper mediastinum.

Several cases of esophagectomy for esophageal cancer developing after previous surgical treatment for achalasia have been reported, using various approaches, including non-thoracotomy, open transthoracic, thoracoabdominal, and thoracoscopic approaches (**Table 1**).^6,10–14)^ The reported cases showed considerable variation in the morphology and distribution of residual esophageal dilatation. Technical difficulties associated with esophageal dilatation, periesophageal adhesions, or prominent vascular structures have been described in some cases.^6,14)^ However, the impact of the anatomical distribution of esophageal dilatation on thoracoscopic exposure has received limited attention. In the present case, the maximum esophageal diameter was 56 mm, with dilatation predominantly located in the upper thoracic esophagus. Although the prone thoracoscopic approach generally provides favorable exposure around the left recurrent laryngeal nerve and facilitates meticulous lymphadenectomy in this region,^15)^ the predominant upper thoracic dilatation restricted the operative workspace around the left recurrent laryngeal nerve and partially offset this technical advantage. This finding suggests that the anatomical distribution of esophageal dilatation, in addition to its degree, may be an important consideration in preoperative assessment. Preoperative CT assessment of the esophageal diameter, tortuosity, and extent of mediastinal occupation may therefore help anticipate technical difficulties and guide operative planning, including the selection of appropriate traction strategies. However, further clinical experience is needed to determine the predictive value of these imaging findings.

**Table 1:** Reported cases of esophagectomy for esophageal cancer after previous surgery for achalasia

Author (Year)	Age Sex	Previous surgery for achalasia	*Interval (years)	Esophageal morphology and dilatation	Cancer characteristics	Approach for esophagectomy	Reconstruction/Route	Technical findings
Murakami et al.^10)^ (1989)	60 F	Surgery, details NR	42	Flask-type, Grade II	AC; EGJ; pT3(SS)N0M0	Non-thoracotomy	Roux-en-Y/NA	Adhesions and hardening around the distal esophagus/EGJ
Yonekawa et al.^11)^ (1993)	50 F	Petrovsky procedure	8	Spindle-type, Grade II; maximum diameter 58 mm before achalasia surgery and 30 mm after surgery	ESCC; upper thoracic; pA3N2M0	Right thoracotomy	Gastric conduit/ Retrosternal	Tracheal invasion; palliative resection
Higa et al.^12)^ (1994)	77 F	Surgery, details NR	30	Sigmoid-type, Grade III; maximum diameter 60 mm in the lower thoracic esophagus	ESCC; upper thoracic; pA2N0M0	Right thoracotomy	Gastric conduit/ Presternal	Thickened mediastinal pleura
Mamiya et al.^13)^ (2008)	43 F	Gastric patch procedure	24	Marked dilatation of the distal esophagus/EGJ	Barrett AC; lower thoracic to abdominal esophagus; pT3N4M0	Right thoracoabdominal approach	Gastric conduit/ Intrathoracic	NR
Yamasaki et al.^6)^ (2017)	68 M	Heller-Dor procedure	33	Dilated esophagus, predominantly in the upper to middle thoracic esophagus	Multiple ESCCs; thoracic esophagus; pT1b(SM2)N0M0	Prone thoracoscopic	Gastric conduit/ Posterior mediastinal	Difficulty securing the operative field due to esophageal dilatation; frequent repositioning of the grasping point
Yankov et al.^14)^ (2025)	70 M	Myotomy with diaphragmatic fundopexy	30	Markedly dilated and tortuous megaesophagus involving the thoracic esophagus	AC; lower thoracic; pT1aN0M0	Right thoracotomy	Gastric conduit/NR	Severe periesophageal and abdominal adhesions; numerous enlarged arterial and venous branches
Present case	73 M	Heller-Dor procedure	15	Straight-type, Grade II; maximum diameter 56 mm in the upper thoracic esophagus	ESCC; middle thoracic; pT1b(SM2)N0M0	Prone thoracoscopic	Gastric conduit/ Retrosternal	Restricted workspace around the left RLN due to upper thoracic dilatation; repositionable traction facilitated exposure

*Interval indicates the time from achalasia surgery to the diagnosis of esophageal cancer.

AC, adenocarcinoma; EGJ, esophagogastric junction; ESCC, esophageal squamous cell carcinoma; F, female; M, male; NA, not applicable; NR, not reported; RLN, recurrent laryngeal nerve

To address this challenge, an internal organ retractor was used to facilitate esophageal traction and operative exposure during mediastinal dissection. As a self-retaining retraction device, the internal organ retractor can provide stable traction without continuous manual assistance and can be repositioned as needed.^16)^ In the present case, the anchoring needle supplied with the device was not used; instead, a 2-0 nylon suture was tied to the retractor and brought extracorporeally through the chest wall to provide traction, similar to a previously described modified marionette technique using the internal organ retractor in thoracoscopic surgery.^17)^ This approach allowed the direction and degree of traction to be adjusted externally according to the operative field. The retractor was applied, whenever possible, to the portion of the esophagus scheduled for resection to minimize the clinical consequences of potential traction-related tissue injury. Nevertheless, excessive traction on a markedly dilated and heavy esophagus may theoretically cause tissue injury, tearing, or bleeding. Therefore, the traction point and tension should be carefully adjusted according to the condition of the esophageal wall and the operative field.

A separate technical consideration was the feasibility of gastric conduit reconstruction after previous Heller-Dor surgery. As summarized in **Table 1**, the stomach was used for reconstruction in several previously reported cases.^6,11–15)^ However, the influence of previous achalasia surgery on gastric conduit reconstruction has been described in limited detail. Preoperative coronal CT demonstrated minimal gastric deformity, with no apparent anatomical distortion that would preclude use of the stomach as a conduit. Based on these findings, the stomach was considered the primary reconstructive organ, and gastric conduit construction was successfully completed despite adhesions from the previous surgery. This experience suggests that careful preoperative assessment of gastric anatomy may be important when considering the stomach as a reconstructive organ after previous surgery for achalasia.

## CONCLUSIONS

In conclusion, esophageal squamous cell carcinoma may develop long after definitive surgical treatment for achalasia, highlighting the importance of long-term endoscopic surveillance even after successful symptom control. In thoracoscopic esophagectomy, both the degree and anatomical distribution of esophageal dilatation may be important considerations, as predominant upper thoracic dilatation may restrict the operative workspace around the left recurrent laryngeal nerve. Appropriate intraoperative traction with flexible repositioning of the traction point may help maintain adequate operative exposure and facilitate safe mediastinal dissection. Furthermore, gastric conduit reconstruction was feasible in the present case despite previous Heller-Dor surgery, although careful preoperative assessment of gastric anatomy may be important when considering this reconstructive option.

## DECLARATIONS

### Availability of data and materials

The data are not publicly available because they contain information that could compromise the privacy of the patient.
